# Regular Medicaid Home Visits and Emergency Department Use Among Older Adults During Extreme Heat

**DOI:** 10.1001/jamanetworkopen.2025.54225

**Published:** 2026-01-15

**Authors:** Hyunjee Kim, Katherine Courchaine, Angela Senders, Clint Sergi, R Tamara Konetzka

**Affiliations:** 1 Center for Health Systems Effectiveness, Oregon Health and Science University, Portland; 2 Biological Sciences Division, Department of Public Health Sciences, University of Chicago, Chicago, Illinois

## Abstract

**Importance:**

Extreme heat poses substantial health risks to older adults. Medicaid home visits in the weeks preceding such events may alleviate these risks.

**Objective:**

To assess whether receiving regular Medicaid home visits prior to extreme heat events is associated with reduced emergency department (ED) visits during extreme heat.

**Design, Setting, and Participants:**

This cohort study used claims and weather data. The changes in daily ED visits during and up to 2 days after extreme heat events were compared between dual-eligible enrollees who received weekly home visits in the preceding month (treatment group) and those who did not (comparison group). Participants included dual-eligible enrollees aged 65 years or older who received at least 1 Medicaid home visit in 2018 or 2019 in zip code tabulation areas (ZCTAs) across the US that experienced at least 1 extreme heat event between May and October in 2018 and 2019. Data were analyzed from June 2024 to October 2025.

**Exposure:**

An extreme heat day was defined at the ZCTA level as a day when the maximum temperature exceeded 90 °F and was at least the 97th percentile for the same day of the year from 2006 to 2017. Consecutive extreme heat days were grouped into extreme heat events when they lasted 1 to 5 days.

**Main Outcome and Measures:**

Daily ED visits per 1000 enrollees.

**Results:**

Our sample included 597 388 dual-eligible enrollees (mean [SD] age, 77.2 [8.2] years; 423 474 female [70.9%]). During extreme heat, daily ED visits per 1000 enrollees increased by 1.29 (95% CI, 1.22 to 1.37) for those who received regular home visits in the preceding month and by 1.25 (95% CI, 1.19 to 1.31) for those who did not, a nonsignificant difference of 0.05 (95% CI, −0.04 to 0.14) between the 2 groups. Similar patterns in stratified analyses were found by climate region and among those with heat-sensitive chronic conditions.

**Conclusions and Relevance:**

In this cohort study of community-dwelling dual-eligible enrollees, regular Medicaid home visits prior to heat events were not associated with changes in ED visits during extreme heat. This finding suggests that while regular home visits may not have reduced ED visits during heat events, they could have helped facilitate timely care-seeking when heat-related symptoms arose.

## Key Points

**Question:**

What is the association between regular Medicaid home visits in the month before extreme heat events and emergency department (ED) visits during heat events among dual-eligible enrollees 65 years or older?

**Findings:**

In this cohort study of 597 388 dual-eligible enrollees, there was an increase in ED visits during extreme heat. The increase in ED visits was similar among dual-eligible enrollees who received regular Medicaid home visits in the preceding month and those who did not.

**Meaning:**

These findings suggest that home visits before a heat event were not associated with reduced ED visits during heat events, but they may have helped facilitate timely care-seeking when heat-related symptoms arose.

## Teaser

This cohort study assesses whether receipt of regular Medicaid home visits in the weeks prior to extreme heat events is associated with reduced emergency department (ED) visits during extreme heat.

## Introduction

Extreme heat events pose a significant health risk.^1,2^ Older adults are particularly susceptible due to age-related physiological changes, such as reduced ability to regulate body temperature and cognitive or functional impairments that may limit their ability to respond effectively to heat. A growing body of evidence links extreme heat with increased hospitalizations, emergency department (ED) visits, and deaths among older adults.^3,4,5^

Dual-eligible enrollees (those enrolled in both Medicare and Medicaid) aged 65 years or older are at particularly high risk for extreme heat. On average, they are more likely to live alone,^6^ experience poverty,^7^ and have poor health,^8^ which are all risk factors for heat vulnerability. Many also receive Medicaid-funded home visits, indicating limitations in activities of daily living that heighten risk during extreme heat.^9^

Although receiving Medicaid home visits signals higher risks, these visits also present an opportunity to mitigate heat-related health outcomes for dual-eligible enrollees. Particularly, regular home visits in the weeks preceding extreme heat may help prevent adverse outcomes. Personal care workers might assess in advance whether homes are adequately cooled and ventilated. They can also provide guidance on heat safety, such as staying hydrated, managing medications appropriately, and avoiding outdoor activities. Alternatively, regular home visits prior to heat events may still lead to ED visits—for example, if personal care workers educate patients on how to identify early signs of heat-related illness. Nevertheless, overall, these visits can play a preventive role in helping older adults avoid heat-related health issues in the first place.

Despite this potential, the role of regular home visits prior to heat events on heat-associated outcomes among dual-eligible enrollees remains unexamined. To address this gap, we evaluated how regular home visits in the month before extreme heat were associated with ED visits among community-dwelling dual-eligible enrollees.

## Methods

The Oregon Health and Science University institutional review board approved this study with a waiver of informed consent because seeking informed consent from all patients included in the study was not feasible and the risk to study participants was minimal. We followed the Strengthening the Reporting of Observational Studies in Epidemiology (STROBE) reporting guideline for cohort studies.

### Data

We used Medicare fee-for-service claims, Medicare Advantage encounter records, and Medicaid claims. Weather data included daily maximum temperature, daily mean relative humidity, and daily mean PM_2.5_ data.^10,11^ Weather variables were aggregated to the zip code tabulation area (ZCTA) level, then converted to zip codes, and linked to each person’s zip code of residence (eTable 1 in Supplement 1).

### Study Sample

We included community-dwelling dual-eligible enrollees aged 65 years or older who resided in the contiguous US between May and October in 2018 and 2019. We required individuals to be enrolled in either Medicare fee-for-service or a Medicare Advantage plan with full Medicaid benefits. We excluded those in Medicare Advantage contracts with high rates of missing data and those enrolled in Medicare for less than 2 years prior to their month of study entry to obtain reliable baseline health information. We further restricted the sample to those who received at least 1 Medicaid-funded home visit at a private home residence (not including assisted living or adult group home) during 2018 and 2019 and to those in ZCTAs that experienced at least 1 heat event during the study period. Lastly, we dropped Utah and Arkansas due to Medicaid data quality concerns. The final sample included 597 388 dual-eligible enrollees in 47 states and Washington DC (eTable 2 in Supplement 1).

### Outcome

Our outcome was whether each dual-eligible enrollee had any all-cause ED visit on a given day.^5^ Additional information can be found in eAppendix 1 in Supplement 1.

### Treatment and Comparison Groups

The treatment group included community-dwelling dual-eligible enrollees who received at least 1 home visit per week during the month prior to the start of an extreme heat event. The comparison group included community-dwelling enrollees who did not receive any home visits during the same 1-month window but had received at least 1 home visit at some point in 2018 or 2019. Because the need for home visits generally reflects greater underlying health risks, both groups were likely to share similar baseline characteristics. To avoid potential confounding, we excluded enrollees who received Medicare-funded home health visits from both groups. While Medicaid home visits include various types of visits, such as personal care, companion visits, meal delivery, and home modifications, we focused on visits involving meaningful interactions between workers and older adults, such as personal care or companion visits (eAppendix 2 in Supplement 1).

### Extreme Heat Event

An extreme heat day for a ZCTA was defined as a day when the maximum temperature exceeded 90 °F and was at least the 97th percentile of the temperature distribution for the same calendar day during the 12 years preceding our study period.^5^ Once extreme heat days were identified, we grouped consecutive days meeting this definition into extreme heat events lasting 1 to 5 days. We dropped heat events lasting longer than 5 days because they were rare.

### Preperiod and Postperiod

We defined the preperiod as the 14 days preceding the start of a heat event. The postperiod included the heat event and the following 2 days. We selected a 2-day follow-up period because the risk of adverse health events remains elevated for multiple days after heat exposure.^3,5^

### Statistical Analysis

We conducted a difference-in-differences analysis, with person-day as the unit of analysis. This approach compared changes in daily ED visits during the heat event and 2 days after for people who received home visits in the preceding month (treatment group) vs those who did not (comparison group).

The regressions included an interaction between the treatment group indicator and the postperiod indicator, as well as separate terms for treatment group and postperiod indicators. The interaction coefficient captured the added effects of regular prior home visits during extreme heat, which was our primary interest. We also included indicators for each ZCTA-specific heat event to ensure we compared treatment and control groups within the same heat event and to account for variation in heat intensity and duration across events. Additionally, we adjusted for individual characteristics, including age, sex, race and ethnicity (Research Triangle Institute categorization), original reason for Medicare entitlement, Medicare plan type, 24 health condition indicators based on Chronic Condition Warehouse algorithm, frailty index score,^12^ and an indicator of having received home visits other than personal or companion visits (eTable 3 in Supplement 1).

We additionally adjusted for the Climate Vulnerability Index’s community baseline social and economic indicator score for each person’s ZCTA,^13^ which captures overall ZCTA-level income, education, housing conditions, and access to transportation. We also included daily ZCTA-level relative humidity and PM_2.5_, and indicators for climate regions defined by the National Centers for Environmental Information, based on each person’s residential ZCTA.^14^ We did not adjust for ZCTA, year, or month indicators because the ZCTA-specific heat event indicators already captured all ZCTA and time variation. Each event was uniquely defined within a single ZCTA and year, making ZCTA and year indicators perfectly collinear. For most events, pre- and postperiods also fell within the same month, making month indicators perfectly collinear as well. We clustered standard errors at the ZCTA level. We used linear regressions for straightforward interpretation of the interaction terms as absolute differences in ED visits. Linear regression results were nearly identical to those from pseudo-Poisson regressions, which can accommodate binary outcomes (eTable 4 in Supplement 1).^15,16^

We included all climate regions in our main regression, but also ran stratified regressions by climate region.^14^ Prior research suggests that the association between extreme heat and adverse outcomes may vary substantially across these regions.^5,17^ The climate regions include Northeast (Connecticut, Delaware, Maine, Maryland, Massachusetts, New Hampshire, New Jersey, New York, Pennsylvania, Rhode Island, and Vermont); Northern Rockies and Plains (Montana, Nebraska, North Dakota, South Dakota, and Wyoming); Northwest (Idaho, Oregon, and Washington); Ohio Valley (Illinois, Indiana, Kentucky, Missouri, Ohio, Tennessee, and West Virginia); South (Arkansas, Kansas, Louisiana, Mississippi, Oklahoma, and Texas); Southeast (Alabama, Florida, Georgia, North Carolina, and South Carolina); Southwest (Arizona, Colorado, New Mexico, and Utah); Upper Midwest (Iowa, Michigan, Minnesota, and Wisconsin); and West (California and Nevada).^14^

We conducted supplemental analyses. First, we stratified the analyses by diagnoses of Alzheimer disease and related dementia, heart failure, chronic kidney disease, and chronic obstructive pulmonary disease. These conditions were prevalent in our study population and increase heat-related health risks, suggesting that those with these conditions may particularly benefit from regular home visits prior to heat events.^18,19,20^

Second, we adjusted for person-specific fixed effects. Some enrollees experienced multiple heat events throughout the study period and their classification as treatment group or comparison group was specific to each heat event. This analysis controlled time-invariant individual characteristics, reducing bias from unobserved confounders.

**Figure 1.**
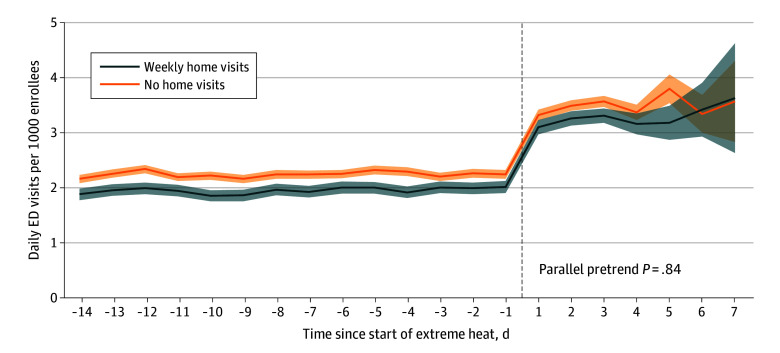
Adjusted Emergency Department (ED) Visits Per 1000 Enrollees During 14 Days Prior to the Start of Extreme Heat, Extreme Heat Event, and 2 Subsequent Days After the End of Extreme Heat Across All Regions, 2018-2019 Shading represent 95% CIs.

The difference-in-differences approach assumes that, without extreme heat, the treatment and comparison groups would have followed parallel trends in ED visits over time, even if baseline rates of ED visits differed (eAppendix 3 in Supplement 1). Although inherently untestable, similarity of pretreatment trends gives credibility to this assumption. This test was not satisfied in 2 climate region subanalyses, the Northwest and Northern Rockies (Figure 1 and eFigures 1 to 9 in Supplement 1). Accordingly, results for these 2 regions should be interpreted with caution, although the pretrends were similar for the national analysis and all other subanalyses.

We used 2-sided *P* < .05 as a threshold for statistical significance. Data were analyzed from June 2024 to October 2025 using R version 4.5.1 (R Project for Statistical Computing) and Stata version 17 (StataCorp).

## Results

Our sample included 597 388 dual-eligible enrollees (mean [SD] age, 77.2 [8.2] years; 423 474 females [70.9%]; 173 914 males [29.1%]). The treatment group was, on average, older (mean [SD] age, 79.2 [8.3] years), more likely to be Black (180 567 [23.6%]) or Hispanic (270 353 [35.3%]), and more frequently enrolled in a Medicare Advantage plan (291 060 [38.0%]) (Table 1). They also had a higher prevalence of chronic conditionst, such as Alzheimer disease and related dementia, chronic kidney conditions, and heart failure. Approximately three-quarters of enrollees with regular home visits before heat events also received a home visit during extreme heat events, while 12 853 (0.9%) of those without regular home visits did. About 0.3% of both treatment (195 [0.03%]) and comparison groups died during heat events (367 [0.03%]).

**Table 1.** Unadjusted Study Sample Characteristics Across All Regions, 2018-2019

Characteristic	Did not receive any home visits during the month prior to a heat event^a^	Received weekly home visits during the month prior to a heat event^b^
No. of enrollee-heat events^c^	1 399 507	765 883
Demographics		
Age, mean (SD), y	76.9 (7.8)	79.2 (8.3)
Sex		
Male	425 723 (30.4)	195 886 (25.6)
Female	973 784 (69.6)	569 997 (74.4)
Race and ethnicity^d^		
American Indian or Alaskan Native	7655 (0.5)	7267 (0.9)
Asian or Pacific Islander	149 058 (10.7)	51 783 (6.8)
Black	287 741 (20.6)	180 567 (23.6)
Hispanic	335 297 (24.0)	270 353 (35.3)
White	587 610 (42.0)	241 560 (31.5)
Unknown	15 951 (1.1)	5823 (0.8)
Other categories	16 195 (1.2)	8530 (1.1)
Medicare status		
Medicare Advantage (vs traditional)	432 481 (30.9)	291 060 (38.0)
Reason for entitlement		
Old age or survivor’s insurance	981 755 (70.2)	534 995 (69.9)
Health status		
Frailty index score, mean (SD)	0.2 (0.1)	0.2 (0.1)
Alzheimer disease or related dementia	191 346 (13.7)	153 952 (20.1)
Arthritis	747 908 (53.4)	459 241 (60.0)
Any cancer	165 281 (11.8)	87 448 (11.4)
Chronic kidney disease	407 524 (29.1)	270 028 (35.3)
Chronic obstructive pulmonary disease	410 044 (29.3)	222 059 (29.0)
Depression	412 785 (29.5)	245 437 (32.0)
Diabetes	732 158 (52.3)	429 061 (56.0)
Heart failure	304 367 (21.7)	198 982 (26.0)
Ischemic heart disease	438 994 (31.4)	265 113 (34.6)
Osteoporosis	246 585 (17.6)	154 208 (20.1)
Parkinson disease	35 683 (2.5)	26 807 (3.5)
Stroke	144 343 (10.3)	95 585 (12.5)
Died during heat-event or 2-d follow-up	367 (0.03)	195 (0.03)
Any personal care visit during heat event	12 853 (0.9)	581 425 (75.9)
Region		
Northeast	588 316 (42.0)	275 499 (36.0)
Northwest	10 101 (0.7)	10 202 (1.3)
Ohio Valley	185 967 (13.3)	99 863 (13.0)
Northern Rockies	9467 (0.7)	1569 (0.2)
South	113 064 (8.1)	161 097 (21.0)
Southeast	261 476 (18.7)	154 820 (20.2)
Southwest	34 738 (2.5)	33 201 (4.3)
Upper Midwest	82 446 (5.9)	18 707 (2.4)
West	113 932 (8.1)	10 925 (1.4)

^a^ Enrollee did not use personal-care HCBS in the 4 weeks prior to heat event.

^b^ Enrollee used personal-care HCBS at least weekly in the 4 weeks prior to heat event.

^c^ Unit of analysis is enrollee-heat event; individual enrollees may contribute more than 1 heat event to analysis.

^d^ Race and ethnicity categories included with the Medicare Beneficiary Summary File RTI_RACE_CD variable. Categories grouped as Other are unavailable to the authors.

Nationwide, the mean (SD) frequency of heat events was 5.2 (2.0) total per ZCTA, varying by region, from 3.3 (1.5) in the Northwest to 6.1 (1.9) in the Southeast (Table 2). The mean length of extreme heat events was 1.8 (0.5) days, ranging from 1.6 (0.4) days in the Northeast to 1.9 (0.5) days in the Northwest, South, and Southwest, and 1.9 (0.6) in the West. The mean (SD) temperature during heat events was 94.0 (3.4) °F, which was 9.5 °F higher than the mean temperature during the 14-day period before the start of heat events.

**Table 2.** Characteristics of Heat Events, 2018-2019

Region	No. of ZCTAs with ≥1 heat event	Mean (SD)			
		No. of heat events per ZCTA	Length of heat event per ZCTA	ZCTA temperature during heat days, F°	ZCTA temperature 14 d prior to heat, F°
All regions	22 491	5.2 (2.0)	1.8 (0.5)	94.0 (3.4)	84.5 (5.0)
Northeast	4604	5.5 (2.2)	1.6 (0.4)	92.4 (1.0)	81.2 (2.0)
Northwest	694	3.3 (1.5)	1.9 (0.6)	94.9 (2.5)	82.4 (4.7)
Ohio Valley	4299	5.2 (1.5)	1.8 (0.5)	92.2 (0.8)	83.1 (2.6)
Northern Rockies	754	3.5 (1.2)	1.6 (0.5)	93.6 (1.6)	78.8 (3.2)
South	3348	4.5 (1.7)	1.9 (0.6)	96.2 (3.3)	89.1 (3.5)
Southeast	3880	6.1 (1.9)	1.8 (0.5)	93.3 (1.2)	87.0 (2.2)
Southwest	879	5.5 (2.1)	1.9 (0.6)	100.3 (6.8)	92.0 (7.6)
Upper Midwest	2487	3.4 (1.3)	1.7 (0.5)	92.4 (1.0)	77.9 (2.3)
West	1546	5.5 (1.9)	1.9 (0.5)	97.9 (4.7)	85.2 (6.7)

Abbreviation: ZCTA, zip code tabulation area.

Among those who received regular home visits, daily ED visits per 1000 enrollees increased from 1.94 (95% CI, 1.91 to 1.98) before heat events to 3.23 (95% CI, 3.17 to 3.30) during heat events and 2 subsequent days, an absolute increase of 1.29 (95% CI, 1.22 to 1.37), representing a 66.5% increase from the preheat period (Table 3). Among those who did not receive home visits, ED visits increased from 2.25 to 3.50, an absolute increase of 1.25 (95% CI, 1.19 to 1.31), corresponding to a 55.6% increase. The difference in the increases between the 2 groups was not statistically significant (0.05; 95% CI, −0.04 to 0.14). These findings are also reflected in Figure 1, which displays adjusted ED visits per 1000 enrollees over time nationwide (eFigure 10 in Supplement 1 for unadjusted rates).

**Table 3.** Daily Emergency Department Visits Per 1000 Enrollees, 2018-2019^a^

Region	Enrollees who received home visits during a month prior to the start of extreme heat			Enrollees who did not receive home visits during a month prior to the start of extreme heat			DiD (95% CI)
	Preheat	Postheat	Post-pre difference	Preheat	Postheat	Post-pre difference	
All	1.94 (1.91 to 1.98)	3.23 (3.17 to 3.30)	1.29 (1.22 to 1.37)	2.25 (2.23 to 2.27)	3.50 (3.44 to 3.55)	1.25 (1.19 to 1.31)	0.05 (−0.04 to 0.14)
Northeast	1.68 (1.63 to 1.73)	2.92 (2.82 to 3.03)	1.24 (1.13 to 1.36)	1.88 (1.85 to 1.90)	3.02 (2.94 to 3.09)	1.14 (1.05 to 1.23)	0.11 (−0.03 to 0.25)
Northwest^b^	2.39 (2.09 to 2.68)	3.70 (3.09 to 4.30)	1.31 (0.64 to 1.98)	3.14 (2.85 to 3.43)	3.87 (3.26 to 4.48)	0.73 (0.05 to 1.40)	0.58 (−0.36 to 1.52)
Ohio Valley	2.32 (2.22 to 2.41)	3.66 (3.47 to 3.85)	1.35 (1.13 to 1.56)	2.80 (2.74 to 2.86)	4.05 (3.91 to 4.19)	1.25 (1.08 to 1.41)	0.10 (−0.17 to 0.36)
Northern Rockies^b^	3.00 (2.07 to 3.94)	4.46 (2.73 to 6.19)	1.46 (−0.54 to 3.46)	2.41 (2.22 to 2.59)	3.28 (2.74 to 3.81)	0.87 (0.22 to 1.51)	0.59 (−1.54 to 2.73)
South	1.97 (1.91 to 2.03)	2.97 (2.85 to 3.09)	1.00 (0.86 to 1.14)	2.22 (2.14 to 2.30)	3.22 (3.05 to 3.39)	1.00 (0.81 to 1.18)	0.00 (−0.22 to 0.23)
Southeast	2.13 (2.04 to 2.22)	3.67 (3.51 to 3.82)	1.54 (1.37 to 1.71)	2.56 (2.51 to 2.61)	3.82 (3.70 to 3.94)	1.27 (1.13 to 1.40)	0.27 (0.07 to 0.48)
Southwest	2.42 (2.26 to 2.58)	3.60 (3.26 to 3.93)	1.18 (0.79 to 1.57)	3.05 (2.90 to 3.19)	4.50 (4.14 to 4.86)	1.45 (1.05 to 1.85)	−0.27 (−0.81 to 0.26)
Upper Midwest	2.26 (2.02 to 2.50)	3.76 (3.27 to 4.25)	1.50 (1.03 to 1.98)	2.49 (2.41 to 2.56)	3.97 (3.76 to 4.17)	1.48 (1.24 to 1.73)	0.02 (−0.50 to 0.54)
West	1.43 (1.06 to 1.81)	2.73 (2.18 to 3.28)	1.30 (0.81 to 1.78)	1.98 (1.93 to 2.04)	3.56 (3.40 to 3.72)	1.58 (1.38 to 1.77)	−0.28 (−0.80 to 0.24)

Abbreviation: DiD, Difference-in-differences estimate; ZCTA, zip code tabulation area.

^a^ Unit of analysis was person-day. The regression models included an interaction term between the treatment group indicator and the postperiod indicator, as well as the main effects of each. We also included indicators for each ZCTA-specific heat period to ensure treatment and control groups were compared within the same heat period and to account for variation in heat intensity and duration. The model adjusted for individual characteristics, including age, sex, race and ethnicity, original reason for Medicare entitlement, Medicare plan type, health condition indicators based on Chronic Condition Warehouse algorithms, frailty index score, and an indicator of having received home visits other than personal or companion visits. We additionally adjusted for the Climate Vulnerability Index’s community baseline social and economic indicator score for each person’s ZCTA, daily ZCTA-level relative humidity and PM_2.5_, and climate region indicators. We clustered standard errors at the ZCTA level.

^b^ Parallel pretrends test *P* < .05. The parallel trends assumption was not supported in the Northwest and Northern Rockies; results for these regions should be interpreted with caution.

**Figure 2.**
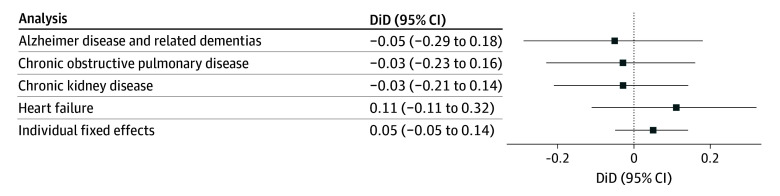
Difference-in-Differences (DiD) Estimates of Daily Emergency Department Visits Per 1000 Enrollees for Subgroups Negative estimates indicate fewer emergency department visits among individuals who received regular home visits prior to a heat event compared to those who did not.

We observed similar patterns across all climate regions (Table 3). An exception was the Southeast, where ED visits increased by 1.54 (95% CI, 1.37 to 1.71) visits for those who had home visits and 1.27 (95% CI, 1.13 to 1.40) visits for those who did not. This resulted in a statistically significant difference of 0.27 (95% CI, 0.07 to 0.48), indicating a greater increase in ED visits among those with home visits. We found that in all supplemental analyses (Figure 2), the increase in ED visits during the heat event and 2 days after did not differ significantly between those with and without home visits.

## Discussion

Linking Medicare and Medicaid claims data from 2018 and 2019 with weather data, we found that dual-eligible enrollees in our study experienced sharp increases in daily ED visits during extreme heat events and the 2 days following. On average, ED visits increased by more than 50% compared with preheat periods, highlighting the substantial health impact of extreme heat. However, when we examined whether home visits mitigated this risk, we found that the increase in ED visits was not significantly different between enrollees who received regular home visits in the month preceding the heat event and those who did not. These patterns remained consistent across nearly all climate regions and among enrollees with heat-sensitive chronic conditions.

Despite broad recognition of extreme heat as a health threat to older adults, evidence on effective interventions beyond air conditioning remains inconclusive.^21,22^ Studies evaluating heat alert systems have shown mixed results, with some reporting null or even a negative impact,^23,24^ while others found associations with reduced mortality in certain cities.^25,26^ Cooling centers may offer protection for high-risk individuals without in-home air conditioning, but current evidence is sparse and lacks causal estimates.^21,27^ As far as we know, no studies have examined the potential protective role of home visits leading up to a heat event in reducing heat-related health risks.

Our findings suggested that regular home visits in the month before an extreme heat event were not associated with changes in ED visits during the heat event. One explanation for these results is that 2 offsetting mechanisms may have been at play. On one hand, home visits before heat events may have helped older adults better prepare for extreme heat by checking cooling systems and reinforcing preventive behaviors, which could reduce the need for emergency care. On the other hand, these visits may also have increased awareness of heat-related symptoms and encouraged timely care-seeking, leading to ED visits when concerning signs appeared. The combination of these opposing effects could have resulted in a similar increase in ED visits during heat events observed in groups with and without regular home visits. Previous studies have similarly found mixed associations. Some found that home visits increased ED visits by identifying clinical issues requiring urgent attention,^28^ and others found reductions in ED visits because home visits effectively addressed care needs in advance.^29^

Ideally, we would assess whether home visits before heat events reduced mortality, because ED visits prompted by early intervention might have led to improved survival. Yet, because we restricted the sample to individuals who survived up to the start of each heat event, we could not evaluate pretrends in mortality, which is critical for a difference-in-differences design. When we compared unadjusted mortality rates during the heat event and the 2 days after, rates were identical (0.03%) for those with and without regular home visits. Yet, without pretrend data, this finding cannot be meaningfully interpreted.

Although the mechanisms underlying our findings remain uncertain, our results raise questions about how Medicaid home visits might support preparation of extreme heat. For example, home care workers could help clients monitor weather forecasts to raise awareness and encourage timely preparations. Future studies could also examine whether incorporating simple heat risk screening (eg, checking in-home cooling resources) or education on safe medication use and hydration improves readiness for heat events. However, even with well-supported home visit programs, there may be inherent limitations to what home visits alone can achieve in reducing health risks of extreme heat. Given the critical role of air conditioning during extreme heat, Medicaid programs could also consider distributing air conditioning units or heat pumps to high-risk populations, or offering utility subsidies to make sure individuals can use cooling devices without fear of prohibitively high electricity bills. Some states have already begun taking this approach.^30,31^ For example, following the deadly 2021 heat wave, Oregon’s Medicaid program began providing air conditioners to eligible enrollees as part of its response to extreme heat events.^31^

It is noteworthy that the Southeast region was the only region where regular home visits were associated with a statistically significant greater increase in ED visits. One explanation is that, because heat events were most frequent in this region, home visits may have heightened awareness of heat-related symptoms and encouraged timely ED care-seeking in response. Alternatively, greater increase in ED visits among home visit users could reflect differences in state Medicaid home visit programs (eg, variation in service scope, visit frequency, or worker training). However, this result should be interpreted cautiously. Although statistically significant, the estimated effect in the Southeast was similar to those in other regions, suggesting that the significance may reflect the region’s larger sample size rather than a substantially different effect.

### Limitations

This study has limitations. First, we could not evaluate the impact of home visits that occurred during the heat event. Approximately three-quarters of individuals with regular home visits prior to a heat event received actual home visits during the heat event, whereas less than 1% of those without prior regular home visits did. However, examining the association between visits during heat events and outcomes presents potential for selection bias. Our data suggested that some visits scheduled during heat events might have been cancelled due to older adults seeking ED care for heat-related symptoms. Furthermore, visits may have been added in response to heat-related concerns or cancelled due to unsafe travel conditions for home care workers.

Second, unmeasured confounding could have biased our estimates. Factors such as the ability to use air conditioning, informal caregiver availability, or neighborhood-level heat mitigation infrastructure (eg, access to cooling centers) could influence the likelihood of ED visits during heat events. Any differences in these factors between treatment and comparison groups could partly explain the overall null findings. Finally, we could not examine mortality as the outcome due to design limitations. Mortality would be an outcome of particular interest for assessing whether regular home visits mitigate the ultimate harm of heat events.

## Conclusions

In summary, we found that regular Medicaid home visits prior to heat events were not associated with changes in ED visits during heat events among older dual-eligible enrollees. While these findings suggest that home visits did not reduce acute care use during extreme heat, they could mean that such visits facilitated appropriate care-seeking when heat-related symptoms arose. Given these mixed possibilities, further research needs to clarify the mechanisms underlying these findings and identify how home visit services can best support older adults during extreme heat. Other policy interventions, such as providing air conditioning units or utility subsidies, may also offer protection. As heat-related health threats continue, it is essential to understand Medicaid’s role in delivering both clinical care and environmental support to high-risk populations.

## Notes

Supplement 1. **eTable 1.** Data sources utilized in this study **eTable 2.** Study sample selection **eAppendix 1.** Identification of emergency department visits **eAppendix 2.** Identification of enrollees who used HCBS in a private home residence **eTable 3.** Additional Medicare variables used in this analysis **eTable 4.** National daily emergency department visits per 1000 enrollees, 2018-2019: Comparison of psuedo Poisson and linear regression models with high dimensional fixed effects **eAppendix 3.** Parallel trends prior to heat event assumption **eFigure 1.** Adjusted emergency department (ED) visits per 1000 enrollees during 14 days prior to the start of extreme heat, extreme heat days, and 2 days after the end of extreme heat in the Northeast, 2018-2019 **eFigure 2.** Adjusted emergency department (ED) visits per 1000 enrollees during 14 days prior to the start of extreme heat, extreme heat days, and 2 days after the end of extreme heat in the Northwest, 2018-2019 **eFigure 3.** Adjusted emergency department (ED) visits per 1000 enrollees during 14 days prior to the start of extreme heat, extreme heat days, and 2 days after the end of extreme heat in the Ohio Valley, 2018-2019 **eFigure 4. **Adjusted emergency department (ED) visits per 1000 enrollees during 14 days prior to the start of extreme heat, extreme heat days, and 2 days after the end of extreme heat in the Northern Rockies, 2018-2019 **eFigure 5. **Adjusted emergency department (ED) visits per 1000 enrollees during 14 days prior to the start of extreme heat, extreme heat days, and 2 days after the end of extreme heat in the South, 2018-2019 **eFigure 6.** Adjusted emergency department (ED) visits per 1000 enrollees during 14 days prior to the start of extreme heat, extreme heat days, and 2 days after the end of extreme heat in the Southeast, 2018-2019 **eFigure 7. **Adjusted emergency department (ED) visits per 1000 enrollees during 14 days prior to the start of extreme heat, extreme heat days, and 2 days after the end of extreme heat in the Southwest, 2018-2019 **eFigure 8. **Adjusted emergency department (ED) visits per 1000 enrollees during 14 days prior to the start of extreme heat, extreme heat days, and 2 days after the end of extreme heat in the Upper Midwest, 2018-2019 **eFigure 9. **Adjusted emergency department (ED) visits per 1000 enrollees during 14 days prior to the start of extreme heat, extreme heat days, and 2 days after the end of extreme heat in the West, 2018-2019 **eFigure 10. **Unadjusted and adjusted emergency department (ED) visits per 1000 enrollees during 14 days prior to the start of extreme heat, extreme heat days, and 2 days after the end of extreme heat in all regions, 2018-2019

Supplement 2.
Data Sharing Statement
