# Supplementary material for: Regular Medicaid Home Visits and Emergency Department Use Among Older Adults During Extreme Heat
Source: JAMA Netw Open. 2026 Jan 15;9(1):e2554225. doi: 10.1001/jamanetworkopen.2025.54225 (PMC12809364; doi:10.1001/jamanetworkopen.2025.54225)
Supplement: Supplement 1. — eTable 1. Data sources utilized in this study eTable 2. Study sample selection eAppendix 1. Identification of emergency department visits eAppendix 2. Identification of enrollees who used HCBS in a private home residence eTable 3. Additional Medicare variables used in this analysis eTable 4. National daily emergency department visits per 1000 enrollees, 2018-2019: Comparison of psuedo Poisson and linear regression models with high dimensional fixed effects eAppendix 3. Parallel trends prior to heat event assumption eFigure 1. Adjusted emergency department (ED) visits per 1000 enrollees during 14 days prior to the start of extreme heat, extreme heat days, and 2 days after the end of extreme heat in the Northeast, 2018-2019 eFigure 2. Adjusted emergency department (ED) visits per 1000 enrollees during 14 days prior to the start of extreme heat, extreme heat days, and 2 days after the end of extreme heat in the Northwest, 2018-2019 eFigure 3. Adjusted emergency department (ED) visits per 1000 enrollees during 14 days prior to the start of extreme heat, extreme heat days, and 2 days after the end of extreme heat in the Ohio Valley, 2018-2019 eFigure 4. Adjusted emergency department (ED) visits per 1000 enrollees during 14 days prior to the start of extreme heat, extreme heat days, and 2 days after the end of extreme heat in the Northern Rockies, 2018-2019 eFigure 5. Adjusted emergency department (ED) visits per 1000 enrollees during 14 days prior to the start of extreme heat, extreme heat days, and 2 days after the end of extreme heat in the South, 2018-2019 eFigure 6. Adjusted emergency department (ED) visits per 1000 enrollees during 14 days prior to the start of extreme heat, extreme heat days, and 2 days after the end of extreme heat in the Southeast, 2018-2019 eFigure 7. Adjusted emergency department (ED) visits per 1000 enrollees during 14 days prior to the start of extreme heat, extreme heat days, and 2 days after the end of extreme heat in the Southwest, 2018-20 [file jamanetwopen-e2554225-s001.pdf]

Supplemental Online Content

Kim H, Courchaine K, Senders A, Sergi C, Konetzka RT. Regular Medicaid home visits and emergency department use among older adults during extreme heat. *JAMA Netw Open*. 2026;9(1):e2554225. doi:10.1001/jamanetworkopen.2025.54225

eTable 1. Data sources utilized in this study

eTable 2. Study sample selection

eAppendix 1. Identification of emergency department visits

eAppendix 2. Identification of enrollees who used HCBS in a private home residence

eTable 3. Additional Medicare variables used in this analysis

eTable 4. National daily emergency department visits per 1000 enrollees, 2018-2019: Comparison of psuedo Poisson and linear regression models with high dimensional fixed effects

eAppendix 3. Parallel trends prior to heat event assumption

eFigure 1. Adjusted emergency department (ED) visits per 1000 enrollees during 14 days prior to the start of extreme heat, extreme heat days, and 2 days after the end of extreme heat in the Northeast, 2018-2019

eFigure 2. Adjusted emergency department (ED) visits per 1000 enrollees during 14 days prior to the start of extreme heat, extreme heat days, and 2 days after the end of extreme heat in the Northwest, 2018-2019

eFigure 3. Adjusted emergency department (ED) visits per 1000 enrollees during 14 days prior to the start of extreme heat, extreme heat days, and 2 days after the end of extreme heat in the Ohio Valley, 2018-2019

eFigure 4. Adjusted emergency department (ED) visits per 1000 enrollees during 14 days prior to the start of extreme heat, extreme heat days, and 2 days after the end of extreme heat in the Northern Rockies, 2018-2019

eFigure 5. Adjusted emergency department (ED) visits per 1000 enrollees during 14 days prior to the start of extreme heat, extreme heat days, and 2 days after the end of extreme heat in the South, 2018-2019

© 2026 Kim H et al. *JAMA Netw Open*.

eFigure 6. Adjusted emergency department (ED) visits per 1000 enrollees during 14 days prior to the start of extreme heat, extreme heat days, and 2 days after the end of extreme heat in the Southeast, 2018-2019

eFigure 7. Adjusted emergency department (ED) visits per 1000 enrollees during 14 days prior to the start of extreme heat, extreme heat days, and 2 days after the end of extreme heat in the Southwest, 2018-2019

eFigure 8. Adjusted emergency department (ED) visits per 1000 enrollees during 14 days prior to the start of extreme heat, extreme heat days, and 2 days after the end of extreme heat in the Upper Midwest, 2018-2019

eFigure 9. Adjusted emergency department (ED) visits per 1000 enrollees during 14 days prior to the start of extreme heat, extreme heat days, and 2 days after the end of extreme heat in the West, 2018-2019

eFigure 10. Unadjusted and adjusted emergency department (ED) visits per 1000 enrollees during 14 days prior to the start of extreme heat, extreme heat days, and 2 days after the end of extreme heat in all regions, 2018-2019

This supplemental material has been provided by the authors to give readers additional information about their work.

© 2026 Kim H et al. *JAMA Netw Open*.

eTable 1. Data Sources utilized in this study

Source	Files	Identification of:	URL
Medicare (Year 2018–2019)	Master Beneficiary Summary File (Base)	Beneficiary age, sex, race, zip code, dual eligibility status, date of death	https://resdac.org/cms-data/files/mbsf-base
Medicare (Year 2018–2019)	Master Beneficiary Summary Files (30 CCW Chronic Conditions Segment)	Chronic conditions	https://resdac.org/cms-data
Medicare (Year 2018–2019)	MedPAR	Emergency department visits	https://resdac.org/cms-data/files/medpar
Medicare (Year 2018–2019)	Inpatient Encounters	Emergency department visits	https://resdac.org/cms-data/files/ip-encounter
Medicare (Year 2018–2019)	Outpatient (Fee-for-Service and Encounters)	Emergency department visits	https://resdac.org/cms-data/files/op-ffs<br>https://resdac.org/cms-data/files/op-encounter
Medicare (Year 2018–2019)	Hospice (Fee-for-Service)	Hospice enrollment date	https://resdac.org/cms-data/files/hospice-ffs
Medicaid T-MSIS Analytic Files (Year 2018–2019)	Demographic and Eligibility Base File	Beneficiary Medicaid eligibility	https://resdac.org/cms-data/files/taf-de
	Other Services File	Beneficiary HCBS utilization	https://resdac.org/cms-data/files/taf-ot
	Long Term Care File	Beneficiary Nursing facility utilization	https://resdac.org/cms-data/files/taf-lt
	Inpatient File	Beneficiary Nursing facility utilization	https://resdac.org/cms-data/files/taf-lt
U.S. Climate Vulnerability Index	Community Baseline Social & Economic Indicator Score	Socioeconomic status	https://climatevulnerabilityindex.org
NOAA		Climate region	https://www.ncei.noaa.gov/access/monitoring/reference-maps/us-climate-regions
Oak Ridge National Laboratory Distributed Active Archive	Daymet: Daily Surface Weather Data on a 1- km grid for North America, Version 4 R1	Temperature, vapor pressure	https://daac.ornl.gov/about/
NASA EarthData	Normalized difference vegetation index (NDVI)	Green space	https://appeears.earthdatacloud.nasa.gov/
Global High-resolution and High-quality Ambient Air Pollutants Dataset over Land		PM2.5	https://zenodo.org/records/10800980

© 2026 Kim H et al. *JAMA Netw Open*.

eTable 2: Study sample selection

Selection Step	Enrollee N									
	All Regions	Northeast	Northwest	Ohio Valley	Rocky Mtns	South	Southeast	Southwest	Upper Midwest	West
Full dual eligible in US	6,083,524	1,555,995	202,435	816,427	61,162	639,864	935,278	250,869	405,014	1,216,480
MA contract with low missingness	5,148,399	1,303,888	180,997	679,805	56,348	591,817	826,252	220,362	321,796	967,134
Enrolled for 2 yrs prior	3,682,222	939,911	124,991	488,876	47,054	432,539	583,255	147,627	238,736	679,233
Has a Medicaid record	3,656,300	933,084	119,103	483,715	46,914	430,890	581,443	146,431	237,135	677,585
Medicaid status is full dual	3,576,237	913,879	116,938	474,129	43,547	429,383	577,660	141,479	205,210	674,012
Consistent Medicare/Medicaid ZIP codes	3,207,567	811,951	93,178	427,064	38,222	390,339	516,238	125,608	181,687	623,280
Not on hospice	3,136,859	802,151	91,410	416,483	37,438	378,159	503,073	123,546	177,511	607,088
State has good TAF quality	3,092,255	802,151	91,410	416,483	37,438	344,753	503,073	112,348	177,511	607,088
No missing CVI score	3,091,905	802,120	91,410	416,454	37,431	344,626	502,986	112,295	177,509	607,074
No missing green space	3,089,241	799,558	91,409	416,444	37,427	344,582	502,973	112,279	177,504	607,065
Used HCBS during study period	1,506,424	445,432	69,894	173,701	13,016	184,478	181,133	46,251	89,311	303,208
Used HCBS in private home	826,604	249,951	18,768	114,014	6,722	124,206	128,571	30,511	56,927	96,934
	**Enrollee-Heat event N**									
Enrollee had ≥ 1 heat event	2,893,607	991,404	30,770	393,107	12,298	431,800	512,608	84,595	122,115	314,910
No Medicare home health pre-heat event	2,526,921	919,613	28,410	334,974	11,821	338,629	447,803	80,008	108,981	256,682
Consistent HCBS use or non-use prior to heat event	2,196,804	875,531	20,568	290,637	11,181	277,505	422,569	68,991	102,733	127,089
Home entire heat event	2,165,390	863,815	20,303	285,830	11,036	274,161	416,296	67,939	101,153	124,857

© 2026 Kim H et al. *JAMA Netw Open*.

eAppendix 1: Identification of emergency department visits

Records for ED visits that result in an inpatient admission are found in the Medicare MedPAR and Inpatient Encounter files and were identified with an Emergency Room Charge Amount of > $0. Otherwise, ED visit claims are located in the Outpatient Fee-for-Service and Encounter files and were identified with revenue center code (REV_CNTR) values of 0450-0459 (emergency room) or 0981 (professional fees-emergency room).[1]

eAppendix 2: Identification of enrollees who used HCBS in a private home residence

We employed a three-step process to identify enrollees who received HCBS that involved a meaningful interaction (e.g. personal care, companion, nursing visit) in a private home. First, we identified HCBS claims for these types of visits in the data. Second, we hierarchically assigned a service setting to each claim: institutional, private home, or unclassifiable. Third, we excluded enrollees from the cohort after the earliest institutional claim was identified. We also excluded enrollees for whom the setting on all HCBS claims was unclassified. Enrollees with a mix of “home” and unclassified” HCBS claims were retained in the final cohort. Details of steps 1 and 2 are described below.

Step 1. Identification of meaningful interaction HCBS claims

We first identified claims for meaningful interaction HCBS using a subset of national and state-specific procedure codes provided in the methodological guidance for *Identifying Home and Community-Based Services and the Enrollees Who Use Them in the TAF* published by CMS.[2]

Procedure codes used to identify meaningful interaction HCBS claims

Procedure Code Type	Procedure Code (Modifier)
National	
CPT	99601*, 99600*, 99509, 99504*, 99503*, 99375*, 99350*, 99349*, 99348*, 99347*, 99345*, 99344*, 99343*, 99342*, 99337*, 99336*, 99335*, 99334*, 99327*, 99326*, 99325*, 99324*
HCPCS	T2025 (GP)*, T2025 (GO)*, T2025 (GN)*, T1022, T1021, T1020, T1019, S9500*, S9131*, S9129*, S9128*, S9127*, S9122, S5181*, S5180*, S5136, S5135, S5131, S5130, S5126, S5125, S5121, S5120, S0274*, S0273*, Q5009*, G0156, G0153*, G0152*, G0151*, T2025 (TD)*, T2025 (TE)*, G0128*, G0154, S9123, S9124, T1000*, T1001*, T1002*, T1003*, T1004*, T1030, T1031, S5110, S5111, S5150, S5151, S9125, T1005
State Specific	
California	Z9525, Z9207, Z9102, Z9028, Z9027, Z8603, Z8595, Z8583, Z8568, Z8567, Z8563, Z8562, Z8561, Z8560, Z8559, Z6920, Z6902, Z6720, Z5838, Z5010, Z5008, Z5002, Z5004, Z5804, Z5806, Z5832, Z5834, Z5836, Z6900, Z9046, Z9047, Z8574, Z8575, Z9029, Z9030, Z9051, Z9053
Colorado	T2025
Connecticut	3022Z, 1554P, 1542P, 1536P, 1535P, 1532P, 1520P, 1225Z, 1214Z, 1212P, 1210Z, 1208Z, 1206Z, 1023Z, 1022Z, 1021Z, 1020Z, 1019Z, 1226Z, 1228Z, 1230Z, 1232Z, 1234Z, 1415Z, 1562P, 3025Z*, 5151C*, 5151D*, 5151E*, 9750Z, 9751Z, 9752Z, 1404Z, 3027Z

© 2026 Kim H et al. *JAMA Netw Open*.

Georgia	T2025 (U5), T2025 (U6), T2025 (HQ), T2025 (UB), S9123, S9124, Y3308, Y3309, Y3310, Y3311, Y3312, Y3313, Y3315, Y3821, Y3826, Y4083
Hawaii	T2025 (UN)*, T2025 (U6)*, T2025 (UP)*, T2025 (U7)*, T2025 (U4)*, T2025 (HM)*, T2025 (UD)*
Idaho	S5121, 8250W, 8247W, 8247A, 8245W, 8245A, 0930T, 0652P, 0649P, 0648P, 0646P, 0643P, 0641P, 0541S, 0541P, 0260B, 0140B, 0150B, 0170B, 0180B, 0190B, 0200B, 0527P, 0528P, 0532P, 0656P, 0657P, 0670P, 0240B*, 0250B*, 0655P, 0810W, 0925T
Iowa	W2518, W2513, W2502, W2501, W2500, W1268, W1267, W1266, W1265, W1208, W1207, W1047, W1040, W1029, W1025, W1003, W2514, W2519, W1027, W1042, W1046, W1050, W1301, W1405, W1420, W2503, W2504, W2505, W3246, W3964
Kansas	T2025
Louisiana	Z0003, Z0013, Z0310
Maryland	W5533, W5532, W5528, W5527, W5521, W5519, W5522, W5529, W1002, W0208, W9304, W9305, W9314, W9315, W2105
Minnesota	T2028 (U1), T1004, T1002, T1003, S5150, S5151, S5126
New Hampshire	T2025 (SE, UC), T2025 (SE, UA, U5), T2025 (SE, UB, U5), T2025 (SE, UC, U3), T2025 (SE, UC, U4)
New Jersey	Z1829, Z1828, Z1824, Z1823, Z1822, Z1821, Z1820, Z1617*, Z1616*, Z1615*, Z1614, Z1613*, Z1612*, Z1611*, Z1610*, Z1605, Z1600, Z1520, Z1435, Z1413, Z1410, Z1295, Z1290, Z1200, Y9846, Y9845, Y9837, Y7445, Y7444, Y7454, Y7455, Z1205, Z1710, Z1715, Z1720, Z1725, Z1730, Z1735, Z1740, Z1745, Y7338, Y7339, Y7344, Y7345, Y7373, Y7447, Y7456, Y7457, Y7458, Y7459, Y7463, Y9849, Z1210*, Z1215, Z1220, Z1225, Z1230*, Z1480, Z1481, Z1482, Z1483, Z1484, Z1485, Z1486, Z1487, Z1490, Z9638, Z9639
New York	4443*, 4435*, 2742, 2681, 2671, 2633, 2632, 2631, 2627, 2626, 2623, 2622, 2611, 2610, 2602, 2601, 2596, 2595, 2594, 2593, 2508, 2507, 2502, 2501, 2424, 2423, 2422, 2406, 2405, 2404, 2403, 2402, 2401, 9799, 2694, 2695, 2825, 4372, 4373, 4763, 4773, 1323, 1324, 3864, 3865, 3906, 3931, 4486*, 4487, 4488, 4489, 4490*, 4491*, 4492, 4493, 4496, 4497, 4498, 4499, 4653*, 4654*, 4655, 4749, 5055, 5056, 9768, 9769, 9770, 9771, 9869, 9875, 4777
North Dakota	W0560
Ohio	T2025
Oregon	SP111, PCA11, OROT1, OR510, ON111, OH111, OC112, OC111, OA111, ECC20, OC115, OR507
Pennsylvania	W7283, W7086, W7085, W7084, W7070, W7069, W7068, W7061, W7060, W7059, W7058, W7057, W6043, W6042, Z9525, W1792, W1729, W1727, W1726, W1725, W1724, W1723, W1701, W1700, W0103, W0101, T2025 (GO), T2025 (GP), T2025 (GN), T2016, T1003, T2025 (TD), T2025 (TE), T2025 (UF), T2025 (UG), T2025 (UJ), T2025 (UH), W1702, W1703, W1705, W1879, W6108, W7062, W7211, W7212, W7213, W7247, W7248, W7249, W7250, W7251, W7252, W7253, W7255, W7256, W7257, W7258, W7261, W7264, W7265, W7266, W7269, W7287, W7288, W7289, W7290, W7301, W7302, W7303, W7304, W8000, W8001, W8002, W8003, W8004, W8005, W8010, W8011, W8012, W8013, W8014, W9593, W9594
South Carolina	T2025
Vermont	T2025 (HW)
West Virginia	W0100, W0101, W0104, W0105
Wyoming	X5136, X5135, X1019, W3071, W3171, W6005, X1005, X2027, X5111

*Additional place of service or program restrictions apply, see HCBS procedure code crosswalk[2]

© 2026 Kim H et al. *JAMA Netw Open*.

Step 2. Identification of service setting on each claim

For each HCBS claim, we used a hierarchical classification scheme to identify the service setting as "institutional", "home", or "unclassified". We defined "institutional" as any residential setting, including but not limited to group homes, adult foster care, residential treatment or habilitation, assisted living facilities, and nursing facilities. Additionally, as a separate step, we searched for claims in the TAF Long-Term file that suggested an enrollee's residence was "institutional". Specific codes are presented in eTables 4 and 4.1.

Hierarchical classification of service setting for HCBS claims

Step	Description	TAF File	Codes	Source	Setting classification
1a	Identify claims for institutional settings using national and state-specific procedure codes (LINE_PRCDR_CD)	OS	**National**: H0008, H0009, H0010, H0011, H0012, H0013, H0017, H0018, H0019, S5140, S5141, T2032, T2033, T2048, T2030, T2031<br>**State-specific**: See table S4.1	HCBS procedure code crosswalk[2]	"Institutional"
1b	On remaining claims, identify claims for institutional settings using benefit type code (BNFT_TYPE_CD)	OS	097, 100, 104	TAF RIF Codebook	"Institutional"
1c	On remaining claims, identify claims for institutional settings using revenue center codes (REV_CNT_CD)	OS	0022, 0024, 0154, 0155, 0156, 0185, 0912, 0913, 1001, 1002	TAF RIF Codebook	"Institutional"
1d	On remaining claims, identify claims for institutional settings using place of service codes (POS_CD)	OS	13, 14, 31, 32, 34, 51, 52, 54, 55, 56, 61	TAF RIF Codebook	"Institutional"
1e	On remaining claims, identify claims for institutional settings using provider taxonomy codes (BLG_PRVDR_TXNMY_CD, SRVC_PRVDR_TXNMY_CD, RFRG_PRVDR_TXNMY_CD, SPRVSNG_PRVDR_TXNMY_CD, DRCTNG_PRVDR_TXNMY_CD)	OS	311500000X, 315D00000X, 315P00000X, 310500000X, 313M00000X, 314000000X, 3140N1450X, 320800000X, 320900000X, 323P00000X, 322D00000X, 320600000X, 320700000X, 324500000X, 3245S0500X, 3104A0625X, 3104A0630X, 310400000X	National Plan and Provider Enumeration System	"Institutional"
1f	On remaining claims, identify claims for home setting using place of service codes (POS_CD)	OS	04, 12	TAF RIF Codebook	"Home"
1g	Remaining claims are deemed unclassified as a service location cannot be determined.	OS	N/A	TAF RIF Codebook	"Unclassified"

© 2026 Kim H et al. *JAMA Netw Open*.

2	Identify claims for institutional long-term care.	LT	Any claim in the LT file where CROSSOVER_CLM_IND=0	TAF RIF Codebook	“Institutional”

State-specific procedure codes for “institutional” setting

**State**	**Procedure codes**
California	Z9103, Z9104, Z9105, Z9106, Z9121, Z9124, Z9032, Z9052
Connecticut	2075Y, 5140X, 5140Y, 5140Z, 9764Z, 9768Z, 9769Z, 9770Z, 1234Z, 1236Z, 1240Z, 1242Z, 1402Z, 9751Z, 9754Z, 9758Z, 9759Z, 1434Z, 1439Z
Delaware	T2025
Georgia	S5150, W9977, Y3600, Y3715
Iowa	W1007, W1009, W1020, W1028, W1043, W1044, W1200, W1201, W1202, W1253, W1254, W1256, W1257, W1259, W1260, W1262, W1263, W1312, W1313, W1314, W1403, W1404, W1405, W2506, W2507, W2508, W2509, W3050, W3963, W2517
Louisiana	HR655, Z0004, Z0014, Z0625
Maryland	W0038, W0039, Z9300, Z9301, W0037, W0226, W0227, W0228, W0229, W0221
New Jersey	Z7333, Y6335, Y7453, Z1230, Z1484, Y7574, Y9633, Y9634, Y9792
New York	1212, 4428, 4430, 4437, 4439, 4440, 4700, 4701, 4702, 4703, 4704, 4705, 4706, 4707, 4708, 4709, 4710, 4711, 4712, 4713, 4714, 4718, 4719, 4722, 4723, 4724, 4725, 2692, 2693, 2820, 2822, 2823, 2834, 2836, 3817, 3826, 3827, 3828, 3829, 3862, 3863, 3946, 3965, 5057, 5058, 5059, 5060, 5061, 5062, 9876, 9877, 9878, 9995
Oregon	AF001, AF002, RC001, SRX01, LF001
Pennsylvania	88UB0124, 88UB0134, 88UB0136, 88UB0144, 88UB0146, 88UB0154, 88UB0156, 88UB0204, W0100, W0102, W6092, W6093, W6094, W6095, W6096, W6097, W6098, W6099, W7022, W7023, W7024, W7025, W7026, W7027, W7028, W7028, W7029, W7030, W7037, W7038, W7039, W7040, W7078, W7079, W7080, W7081, W7082, W7083, W7233, W7234, W7291, W7292, W7293, W7294, W7295, W7296, W7297, W7298, W0604, W0616, W1704, W1705, W7259, W7260, W7262, W7263, W7267, W7268, W7270, W7299, W7300
West Virginia	W1682
Wyoming	W4000, W6006

© 2026 Kim H et al. *JAMA Netw Open*.

eTable 3. Additional Medicare variables used in this analysis

Variable	Medicare Fee-for-Service Files	Variables Used to Construct
Demographics		
Age	MBSF Base	bene_birth_dt
Race/Ethnicity	MBSF Base	rti_race_cd
Sex	MBSF Base	sex
Original reason for Medicare entitlement	MBSF Base	orec
Health characteristics		
Acute myocardial infarction	MBSF CCW30	ami
Alzheimer's or related dementia	MBSF CCW30	alzhdmta/alzh, nonalzh_demen
Arthritis	MBSF CCW30	ra_oa
Asthma	MBSF CCW30	asthma
Arial fibrillation	MBSF CCW30	atrialfb/atrial_fib
Benign prostatic hyperplasia	MBSF CCW30	hyperp/bph
Cancer	MBSF CCW30	cncrbrst, cncrclrc, cncrendm, cncrlung, cncrbprst/ cancer_breast. cancer_colorectal, cancer_endometrial, cancer_lung, cancer_prostate
Cataract(s)	MBSF CCW30	cataract
Chronic kidney disease	MBSF CCW30	chronickidney
Chronic obstructive pulmonary disease	MBSF CCW30	copd,
Depression	MBSF CCW30	depress/depression
Diabetes	MBSF CCW30	diabetes
Glaucoma	MBSF CCW30	glaucoma
Heart failure	MBSF CCW30	chf/hf
Hip fracture	MBSF CCW30	hipfrac/hip_fracture
Hyperlipidemia	MBSF CCW30	hyperl/hlp
Hypertension	MBSF CCW30	hypert/htn
Hypothyroid	MBSF CCW30	hypoth/hypthyrd
Ischemic heart disease	MBSF CCW30	ischmcht/ischemicheart
Osteoporosis	MBSF CCW30	osteoprs/osteoporosis
Stroke/transient ischemic attack	MBSF CCW30	strketia/stroke_tia
First hospice claim date	MBSF CCW30	hspcstrt
Frailty index score*	MedPAR or IP, OP, Carrier, HHA, and DME	icd_dgns_cd_xx, hcpcs_cd

Note: Additional covariables used in this analysis are included in eTable 1.

*Frailty index created by Kim et al.[3]

© 2026 Kim H et al. *JAMA Netw Open*.

eTable 4. National daily emergency department visits per 1000 enrollees, 2018-2019: Comparison of pseudo-Poisson and linear regression models with high dimensional fixed effects.

	Enrollees who used Home Visits in the month before extreme heat			Enrollees who did not use Home Visits in the month before extreme heat			
**Model**	**Pre-Heat**	**Post-Heat**	**Post-Pre Difference**	**Pre-Heat**	**Post-Heat**	**Post-Pre Difference**	**DiD (95% CI)**
Psuedo Poisson (ppmlhdfe)	2.47 (2.43, 2.51)	3.94 (3.86, 4.02)	1.47 (1.38, 1.55)	2.78 (2.75, 2.80)	4.36 (4.30, 4.42)	1.58 (1.51, 1.65)	-0.12 (-0.22, -0.01)
Linear probability (reghdfe)*	2.44 (2.40, 2.48)	3.98 (3.90, 4.07)	1.55 (1.45, 1.64)	2.79 (2.76, 2.81)	4.33 (4.26, 4.41)	1.55 (1.46, 1.63)	0.001 (-0.11, 0.12)

DiD: Difference-in-differences

* The ppmlhdfe Poisson regression drops observations with no variation in the outcome or where cells exhibit separation, whereas the reghdfe linear model retains them. To facilitate model comparison, the linear estimates presented here were calculated on the same estimation sample used by ppmlhdfe.

Note: Unit of analysis was person-day. The regression models included an interaction term between the treatment group indicator and the post-period indicator, as well as the main effects of each. We also included indicators for each ZCTA-specific heat period to ensure treatment and control groups were compared within the same heat period and to account for variation in heat intensity and duration. The model adjusted for individual characteristics, including age, sex, race and ethnicity, original reason for Medicare entitlement, Medicare plan type, health condition indicators based on Chronic Condition Warehouse algorithms, frailty index score, and an indicator of having received home visits other than personal or companion visits. We additionally adjusted for the Climate Vulnerability Index’s community baseline social & economic indicator score for each person’s ZCTA, daily ZCTA-level relative humidity and PM2.5. We clustered standard errors at the ZCTA level.

© 2026 Kim H et al. *JAMA Netw Open*.

eAppendix 3. Parallel trends prior to heat event assumption

To assess the parallel trends assumption, we limited the sample to enrollee-days during the pre-heat event and compared adjusted outcomes between treatment and comparison groups on each day from 2 to 14 days before the heat event, using day 1 (the day immediately before the heat event) as the reference period. If no significant differences in outcomes were observed between groups on days 2–14 relative to day 1, the parallel trends assumption would be supported. The unit of analysis was the enrollee-day. We conducted ordinary least squares regression, modeling the outcome as a function of treatment group indicator, day indicators (with day 1 as reference), and interactions between treatment group and day indicators. Models adjusted for the same independent variables used in the main analysis, including a fixed effect for heat event, and clustered standard errors on ZCTA. Results of the parallel trends assumption test are shown in Figures 1-2 and eFigures 1-9.

eFigure 1. Adjusted emergency department (ED) visits per 1000 enrollees during 14 days prior to the start of extreme heat, extreme heat days, and 2 days after the end of extreme heat in the Northeast, 2018-2019. Horizontal dotted lines represent 95% confidence intervals.

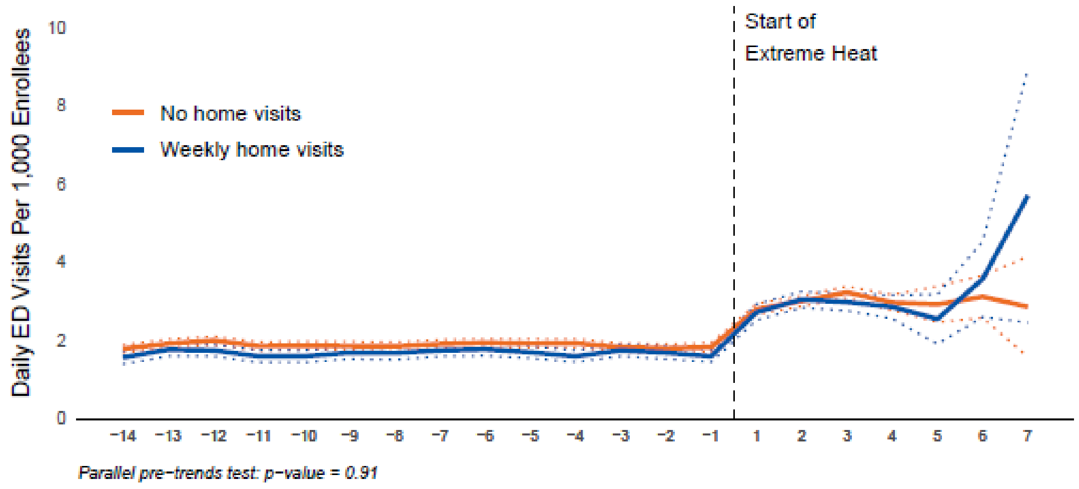

eFigure 2. Adjusted emergency department (ED) visits per 1000 enrollees during 14 days prior to the start of extreme heat, extreme heat days, and 2 days after the end of extreme heat in the Northwest, 2018-2019. Horizontal dotted lines represent 95% confidence intervals.

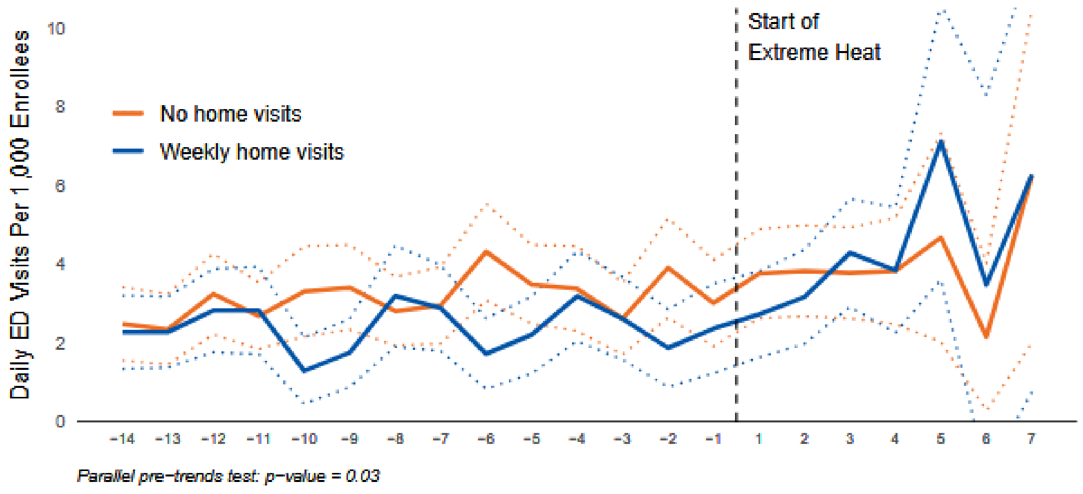

© 2026 Kim H et al. *JAMA Netw Open*.

eFigure 3. Adjusted emergency department (ED) visits per 1000 enrollees during 14 days prior to the start of extreme heat, extreme heat days, and 2 days after the end of extreme heat in the Ohio Valley, 2018-2019. Horizontal dotted lines represent 95% confidence intervals.

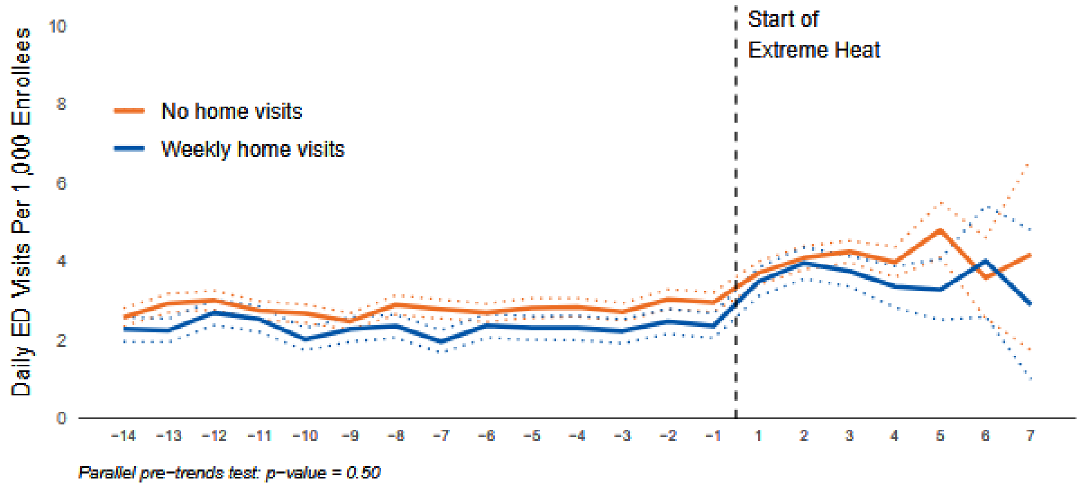

eFigure 4. Adjusted emergency department (ED) visits per 1000 enrollees during 14 days prior to the start of extreme heat, extreme heat days, and 2 days after the end of extreme heat in the Northern Rockies, 2018-2019. Horizontal dotted lines represent 95% confidence intervals.

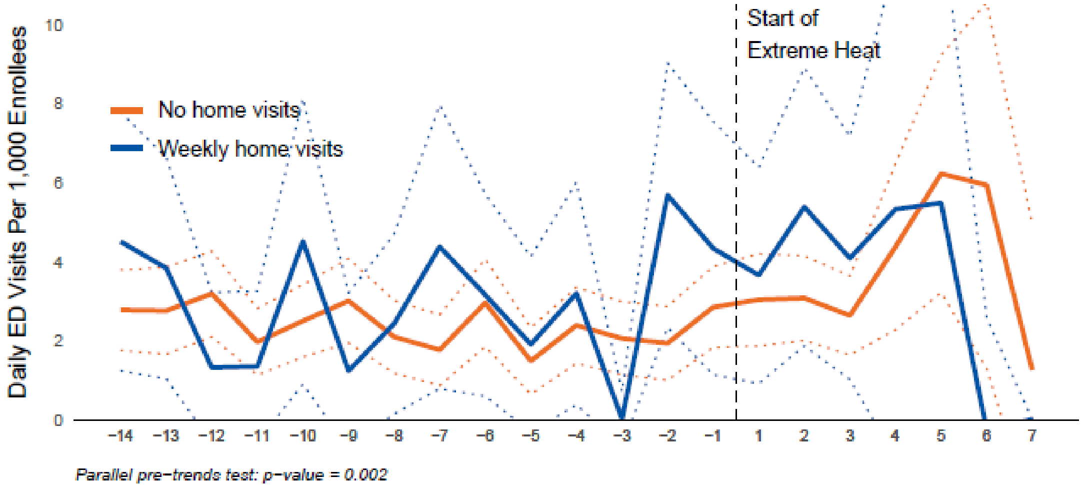

© 2026 Kim H et al. *JAMA Netw Open*.

eFigure 5. Adjusted emergency department (ED) visits per 1000 enrollees during 14 days prior to the start of extreme heat, extreme heat days, and 2 days after the end of extreme heat in the South, 2018-2019. Horizontal dotted lines represent 95% confidence intervals.

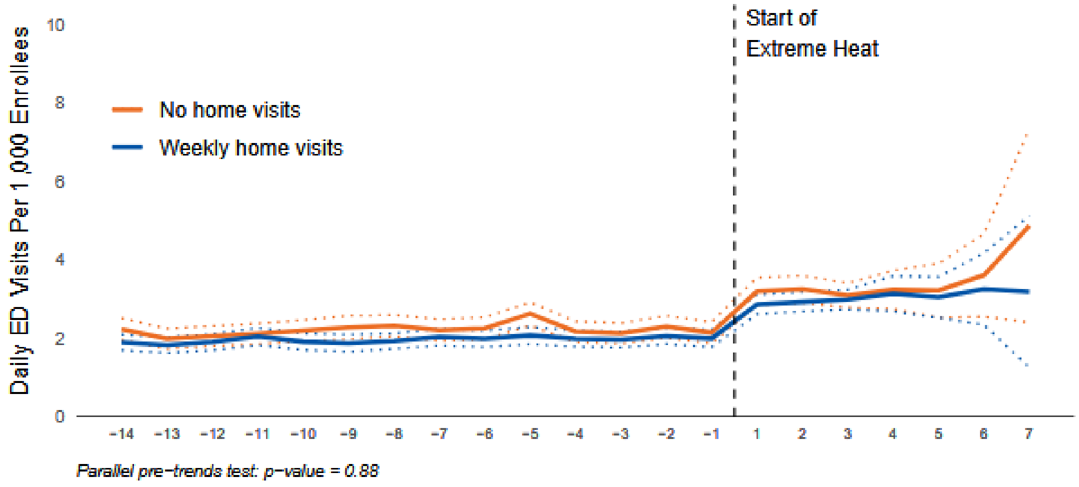

eFigure 6. Adjusted emergency department (ED) visits per 1000 enrollees during 14 days prior to the start of extreme heat, extreme heat days, and 2 days after the end of extreme heat in the Southeast, 2018-2019. Horizontal dotted lines represent 95% confidence intervals.

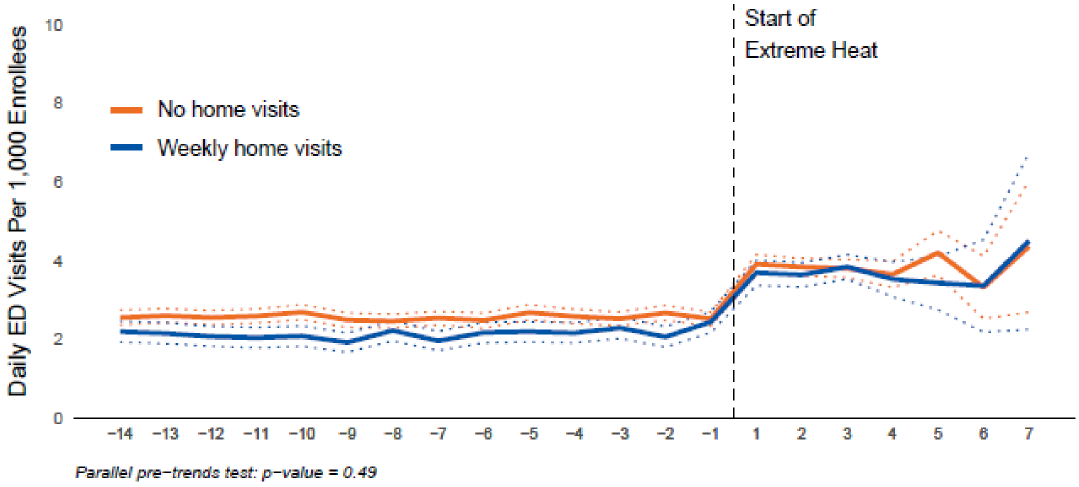

© 2026 Kim H et al. *JAMA Netw Open*.

eFigure 7. Adjusted emergency department (ED) visits per 1000 enrollees during 14 days prior to the start of extreme heat, extreme heat days, and 2 days after the end of extreme heat in the Southwest, 2018-2019. Horizontal dotted lines represent 95% confidence intervals.

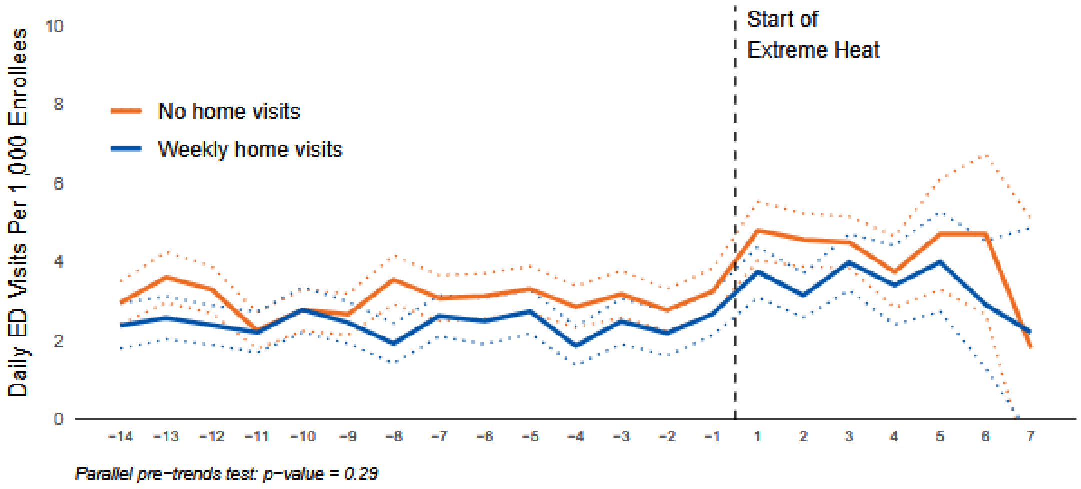

eFigure 8. Adjusted emergency department (ED) visits per 1000 enrollees during 14 days prior to the start of extreme heat, extreme heat days, and 2 days after the end of extreme heat in the Upper Midwest, 2018-2019. Horizontal dotted lines represent 95% confidence intervals.

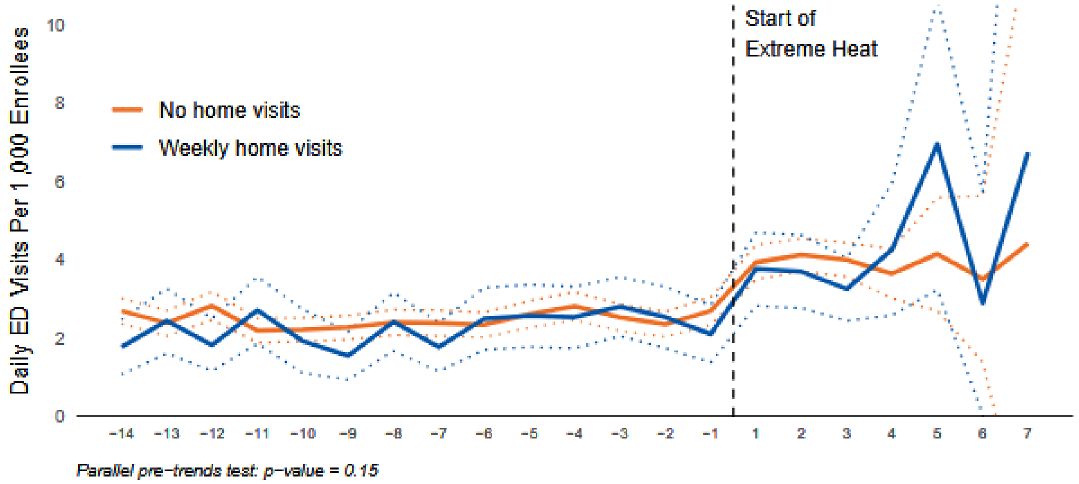

© 2026 Kim H et al. *JAMA Netw Open*.

eFigure 9. Adjusted emergency department (ED) visits per 1000 enrollees during 14 days prior to the start of extreme heat, extreme heat days, and 2 days after the end of extreme heat in the West, 2018-2019. Horizontal dotted lines represent 95% confidence intervals.

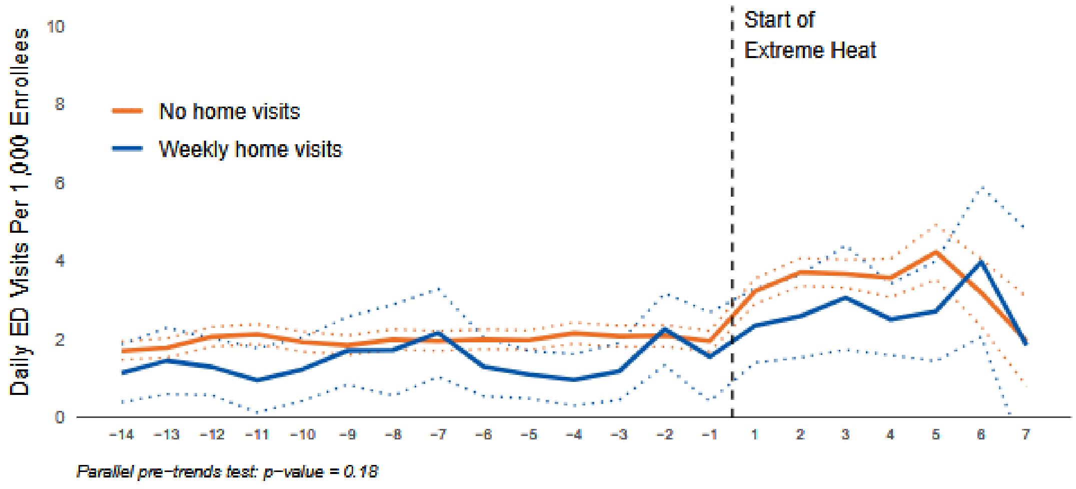

eFigure 10. Unadjusted and adjusted emergency department (ED) visits per 1000 enrollees during 14 days prior to the start of extreme heat, extreme heat days, and 2 days after the end of extreme heat in all regions, 2018-2019. Horizontal dotted lines represent 95% confidence intervals.

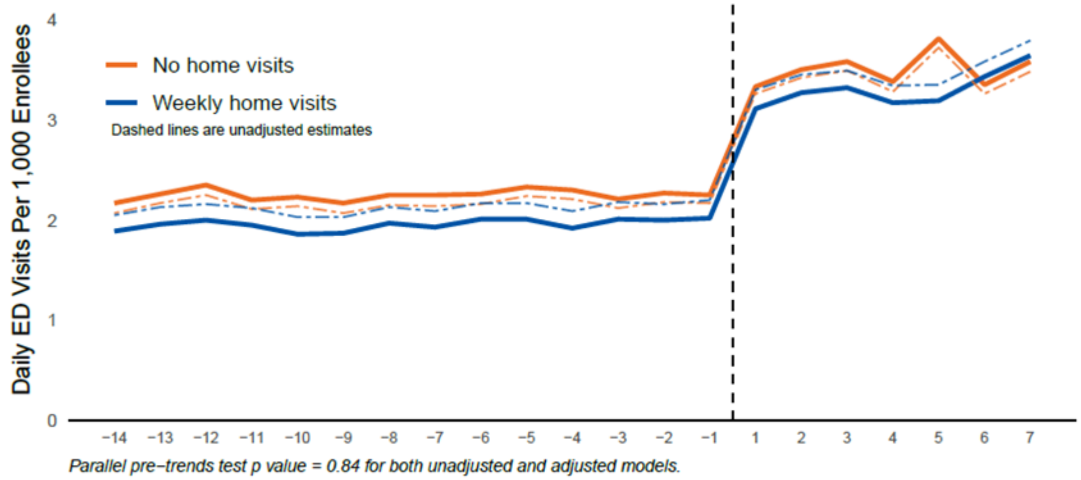

© 2026 Kim H et al. *JAMA Netw Open*.

References

1. How to Identify Hospital Claims for Emergency Room Visits in the Medicare Claims Data | ResDAC. Accessed October 7, 2024. https://resdac.org/articles/how-identify-hospital-claims-emergency-room-visits-medicare-claims-data

2. Greener E, Carpenter A, Nolan L. *Identifying Home and Community-Based Services and the Enrollees Who Use Them in the TAF*. CMS; 2023.

3. Kim DH, Schneeweiss S, Glynn RJ, Lipsitz LA, Rockwood K, Avorn J. Measuring Frailty in Medicare Data: Development and Validation of a Claims-Based Frailty Index. *J Gerontol Ser A*. 2018;73(7):980-987. doi:10.1093/gerona/glx229

© 2026 Kim H et al. *JAMA Netw Open*.
