# Supplementary material for: Regular Medicaid Home Visits and Emergency Department Use Among Older Adults During Extreme Heat
Source: JAMA Netw Open. 2026 Jan 15;9(1):e2554225. doi: 10.1001/jamanetworkopen.2025.54225 (PMC12809364; doi:10.1001/jamanetworkopen.2025.54225)
Supplement: Supplement 2. — Data Sharing Statement [file jamanetwopen-e2554225-s002.pdf]

Data Sharing Statement

Data
Data available: No

Additional Information
Explanation for why data not available: CMS policy prohibits researchers from sharing data sources with anyone not included on the data use agreement.
